# Association between Consumption of Ultra-Processed Foods and Sociodemographic Characteristics in Brazilian Adolescents

**DOI:** 10.3390/nu15092027

**Published:** 2023-04-23

**Authors:** Hélida Ventura Barbosa Gonçalves, Letícia Spricido Batista, Ana Laura Benevenuto de Amorim, Daniel Henrique Bandoni

**Affiliations:** 1 Instituto de Saúde e Sociedade, Universidade Federal de São Paulo, Santos 11015-020, SP, Brazil; 2 Serviço Nacional de Aprendizagem Comercial, Senac Santos, Santos 11015-003, SP, Brazil; 3 Campus Rosinha Viegas, Universidade Metropolitana de Santos, Santos 11045-002, SP, Brazil

**Keywords:** adolescents, ultra-processed foods, food consumption

## Abstract

Background: The consumption of ultra-processed foods is associated with several negative health outcomes. Studies on adolescents have shown that this population has a high consumption of these foods, especially in high-income countries. However, there are no studies on the types of ultra-processed foods consumed. The present study evaluated secondary data from a representative sample of the National School Health Survey, the consumption of ultra-processed foods by 159,245 Brazilian adolescents. Methods: Data were collected via a self-administered questionnaire using a mobile device. A Poisson regression model was used to assess the prevalence of ultra-processed food consumption and its correlation with sociodemographic characteristics. Results: The consumption of ultra-processed foods was significant among Brazilian adolescents, and almost half of the participants reported consumption the day before. We observed that sociodemographic characteristics such as school type, race/skin color, region, municipality type, age, living with mother, living with father, and maternal education level were associated with greater or lesser consumption of ultra-processed foods. Adolescents who study in private schools, are female, white, and live in non-capital cities consume more ultra-processed foods. Conclusions: Access to in natura and minimally processed foods must be on the agenda of governments and encouraged by food and nutrition education to guarantee the right to adequate and healthy food.

## 1. Introduction

The global rise in overweight and nutrition-related non-communicable diseases (NCDs) is related to changes in the pattern of food consumption and physical inactivity [1]. A major concern has been the impact of ultra-processed foods (UPFs) and ultra-processed drinks on weight gain and the risk of NCDs at all stages of life [2,3,4]. 

The definition of industrialized products limits the knowledge of the industrial processing levels of food, hindering food choices. In 2016, the NOVA classification divided foods into four different groups according to the extent and purpose of the processing to which they are subjected, giving rise to four groups: in natura, minimally processed, processed, and ultra processed [5]. Ultra-processed foods are processed food substances that may contain little or no whole food, and usually include flavorings, colorings, emulsifiers, and other food additives. In addition to being attractive from the point of view of palatability, they are generally inexpensive and highly profitable for transnational food industries [6]. The new classification was incorporated into the food guide for the Brazilian population, which brings the reflection that is preferred for consumption in natura and/or minimally processed foods, controls the consumption of processed foods, and avoids the consumption of ultra-processed foods as a result of being excessive calorie foods that have an the impact on the environment [7]. Furthermore, ultra processed is different from beneficial food processing, which helps preserve the nutrient content and increase food safety, without using unhealthy additives [6].

Adolescence is an extremely sensitive period that can change pre-existing habits and develop unhealthy behavioral patterns, such as spending long periods of time in front of screens, consuming meals outside the home [8], and not having meals with the family [9]. These habits can determine a person’s lifestyle and health in the present and the future [10]. In addition, the high consumption of ultra-processed foods in this age group demonstrates a potentially harmful effect on health and often contributes to the impairment of the growth and development of adolescents, which also affects them in adulthood [11]. Studies have reported that children and adolescents are the leading consumers of ultra-processed food in some high-income countries, accounting for over 50% of their total calories [1,2,3,4]. In low- and middle-income countries, the consumption of ultra-processed foods by adolescents is lower than that in high-income countries (less than 40%) [11,12,13]. 

In Brazil, the contribution of ultra-processed foods to the total calorie intake increases among individuals with a higher economic status [14]. However, even among the beneficiaries of income transfer programs, the consumption of ultra-processed foods was reported by 80%. The reasons for consumption were taste (46%), price (24%), and practicality (17%) [15].

A study that analyzed the evolution of food consumption among Brazilian adolescents, students in the last years of elementary school, showed a decreased consumption of unhealthy eating markers (most ultra-processed foods) and an increase in regular vegetable consumption [16]. Also in Brazil, a study based on a specific group of adolescents showed that the consumption of processed foods in the diet of adolescents represented 31.9% of their daily energy consumption [11]. However, there are no data that analyzed the consumption of ultra-processed foods in a nationally representative sample of adolescents, using food consumption reports from the previous day, that is, the previous 24 h.

Ninety percent of adolescents live in low- and middle-income countries, where the barriers to achieving health and support for healthy development are often the most complex and challenging [17]. Thus, the World Health Organization (WHO) recommends the implementation and maintenance of surveillance systems for risk factors affecting the health of adolescents. The main surveys aimed at this population are the Global School-based Student Health Survey (GSHS), Health Behavior in School-aged Children (HBSC), Youth Risk Behavior Surveillance System, and National School Health Survey (PeNSE) in Brazil [18].

Therefore, the objective of this study was to evaluate the consumption of ultra-processed foods by Brazilian adolescents enrolled in public and private schools by analyzing secondary data from PeNSE.

## 2. Materials and Methods

### 2.1. Database and Sample

The study’s data come from the PeNSE [19] of 2019, which represents mostly adolescents from 13 to 17 years of age, made up of students in the final years of elementary and high school. 

Four editions of PeNSE have been performed: 2009, 2012, 2015, and 2019. PeNSE addressed issues related to socioeconomic information, social and family context, drug experimentation and consumption, sexual and reproductive health, violence, safety and accidents, perception of body image, and questions about food consumption and food behavior [19]. Access to these databases is publicly available on the Brazilian Institute of Geography and Statistics (IBGE) website. Therefore, this study used secondary data. The IBGE is the main provider of geographical information and statistics in Brazil.

PeNSE estimates the population parameters for the following geographic levels: Brazil, regions, states, and capital cities. The stratification of the sample groups the schools according to regions and, based on this, selects the students through cluster sampling in two stages: school selection and class selection. Students from the selected classes who were present on a given day were invited to answer the questionnaire, provided they agreed with the Free and Informed Consent Form. 

A total of 159,245 students from 4253 schools were interviewed in this study. Before PeNSE released the data to the general population, a treatment of the database and specification of the fields without the registration of the questionnaires was carried out, such as not answering the questions to follow the filling out and of completing the questionnaire, early abandonment of the questionnaire, and the absence of a response. These situations received different coding in the database but were not analyzed in the present study. Finally, with validated information, 4242 schools and 125,123 students had their questionnaires analyzed as part of this study. 

### 2.2. Data Collection and Study Variables

Data were collected via a self-administered questionnaire using a mobile device. Data were collected through a self-administered structured questionnaire using a mobile collection device (MCD), which corresponds to a smartphone. This device was made available to adolescents by the IBGE exclusively for answering the questionnaire.

The sociodemographic characteristics used for the analyses were: school type (public or private); gender (male and female); race/skin color (white, black, Asian, mixed race, indigenous); region (North, Northeast, Southeast, South, and Midwest); type of municipality (capital and non-capital); age (less than 13 years old; 13–15 years old; 16–17 years old; 18 years old or more); living with mother (yes or no); living with father (yes or no); and maternal education level (no education, incomplete elementary school, complete elementary school, incomplete high school, complete high school, incomplete higher education, and complete higher education). 

The variables of interest in this study were related to the consumption of ultra-processed foods or consumption on the day before the survey. The data was obtained, as indicated by this example: “Yesterday, did you drink fruit juice in a box or can?” The possible answers were “yes” or “no.” 

The following ultra-processed foods were used to assess the consumption on the previous day: 1, crackers, packaged snacks (chips); 2, cookies, cookies with fillings, or packaged cakes; 3, bread, hotdog buns, hamburger buns; 4, soft drinks; 5, margarine; 6, hot dogs, linguiça, bologna, or ham; 7, chocolate, ice cream, gelatin, flan, or industrialized desserts; 8, mayonnaise, ketchup, or other industrialized sauces; 9, chocolate drinks; 10, powdered refreshments; 11, industrialized fruit juice (from a box or can); 12, instant noodles, soup packets, frozen lasagna, or other frozen ready-made foods; and 13, flavored yogurts. 

To make the tables easier to read, the groups were named into: 1, crackers; 2, cookies; 3, bread; 4, soft drinks; 5, margarine; 6, sausages; 7, industrialized desserts; 8, industrialized sauces; 9, chocolate drinks; 10, powdered refreshments; 11, boxed/canned juice, 12, ready-made meals; and 13, flavored yogurts. 

### 2.3. Data Analysis

The prevalence was presented as proportions (%), with a confidence interval of 95% (95% CI) of the consumption of ultra-processed foods stratified by the sociodemographic variables. A Poisson regression model was used to assess the prevalence of consumption of ultra-processed foods and its correlation with the other variables, adjusted for school type, race/skin color, region, type of municipality, age, living with mother, living with father, and maternal education level. We used Stata software version 13.1 (StataCorp LP, College Station, TX, USA) for data analysis, considering the complex design of our sample (cluster). 

### 2.4. Ethical Aspects

PeNSE was approved through an opinion issued by the National Research Ethics Committee of the National Health Council (Conselho Nacional de Saúde—CONEP) no. 3,249,268 on 9 April 2019. 

## 3. Results

The analytical sample of this study consisted of 159,245 Brazilian adolescents for region, administrative dependence of the school, and type of municipality. For other variables, *n* was smaller. The lowest number of responses was for race/skin color, with 155,806 responses. In total, 85.1% of them studied in public schools, 77.2% of them resided in municipalities that were not capital cities, 51.3% were between 13 and 15 years old, and 87.8% lived with their mothers (Table 1). 

Table 2 describes the frequency of consumption of each subgroup of ultra-processed foods the day before the interview. Almost half of the participants reported having consumed crackers (49.6%) and cookies (46.7%), and slightly less than half reported having consumed bread (41.8%), soft drinks (40.5%), and margarine (40.1%). Approximately one-third of the participants reported consuming industrialized desserts (33.3%). Between 20% and 30% of the sample reported the consumption of boxed or canned juice (24.9%), powdered refreshments (24.4%), industrialized sauces (29.5%), or ready-made meals (21.0%). Less than 20% of the participants reported consuming foods from the flavored yogurt subgroup the day before the interview. 

Table 3, Table 4 and Table 5 present the adjusted prevalence ratios for the relationship between the consumption of ultra-processed foods on the previous day and the sociodemographic characteristics. Regarding the types of schools, for students enrolled in private schools, the chances of consuming crackers, cookies, soft drinks, margarine, boxed or canned juice, powdered soft drinks, and flavored yogurts was greater. However, these students were less likely to consume bread, industrialized desserts, and chocolate drinks. Non-capital students have a greater chance of consuming bread, soft drinks, margarine, sausages, industrialized desserts, chocolate drinks, and flavored yogurts than adolescents living in capitals.

Female adolescents were more likely to consume cookies, bread, soft drinks, sausages, chocolate drinks, and flavored yogurts, while male adolescents were more likely to consume margarine and industrialized desserts. 

Black, Asian, mixed-race, and indigenous adolescents were more likely to consume industrialized desserts than white adolescents, but these groups were less likely to consume boxed or canned juice and powdered refreshments compared to white adolescents. Adolescents over 13 years of age were more likely to consume crackers, chocolate drinks, and flavored yogurts, and less likely to consume industrialized sauces. Residents in the Southeast, South, and Midwest regions were less likely to consume soft drinks, industrialized desserts, industrialized sauces, boxed or canned juice, and powdered soft drinks than those in the North region. 

Living with a father led to greater consumption of industrialized desserts and chocolate drinks and less margarine, boxed or canned juice, powdered refreshments, and ready-made meals. In contrast, those living with their mothers were less likely to consume soft drinks. Regarding maternal education level, there was lower consumption of bread, soft drinks, margarine, sausages, industrialized desserts, and chocolate drinks when mothers had no education. 

## 4. Discussion

The ultra-processed foods most consumed by Brazilian adolescents were crackers and cookies (almost half reported consumption on the previous day), followed by bread and soft drinks. The associations between UPF consumption and various sociodemographic characteristics of adolescents were identified in this study. We observed that white female adolescents who studied in private schools and lived in non-capital cities were more likely to consume ultra-processed foods. 

A study that evaluated variations in adolescent food consumption over time from 2009 to 2015 of foods that are markers of healthy and unhealthy eating habits found high proportions of adolescents who regularly consumed (≥5 days/week) candy, soft drinks, and snacks [16]. However, the authors also observed an increase in the consumption of snacks and a decrease in the consumption of soft drinks and candy during the years studied (−7410 and −4407, respectively) [16], corroborating the results of this study, which showed that snacks were the ultra-processed foods with the greatest consumption. In 2009, PeNSE identified the consumption of candy, soft drinks, cookies, and sausages, and found high proportions of consumption of 50.9%, 37.2%, 33.6%, and 18%, respectively [20]. These data were corroborated by Costa et al. (2018) [21], who evaluated the 2015 National School Health Survey and found that approximately 40% of adolescents reported daily consumption of at least one UPF subgroup (39.7%). In the present study, we did not assess the frequency of UPF consumption.

In previous editions of the National School Health Survey [22,23,24], questions were asked about the consumption of industrialized/ultra-processed foods as a marker of unhealthy eating habits; however, there were no details regarding the foods consumed. The inclusion of questions about ultra-processed foods commonly consumed in the 2019 edition made it possible to assess which products were most consumed by adolescents, as well as to verify whether there is differentiation for other audiences. Research carried out with a sample of the Brazilian adult population named the National Research of Health (Pesquisa Nacional de Saúde) found that bread, margarine, and soft drinks were the most frequently consumed ultra-processed products [25]. In 2021, a Brazilian survey found that 18.2% of the study participants consumed five or more groups of ultra-processed foods the day before the interview, and that this indicator showed a tendency to decrease with advancing age [26]. In our study, crackers and cookies were most frequently reported by Brazilian adolescents. This result may be due to the influence of media, given the greater susceptibility of this group to its influence. The consumption of ultra-processed foods is associated with the time spent in front of a television, computer, or cellphone screen [27], which is high among adolescents; this may also explain the decrease in UPF consumption with age [26].

We observed that girls were more likely to consume cookies, bread, soft drinks, sausages, chocolate drinks, and flavored yogurt. In a previous study, it was found that girls, compared to boys, had a positive association with soft drinks, candy, fried snacks, and ultra-processed salty snacks [28]. 

Residents in the Southeast, South, and Midwest regions are less likely to consume soft drinks, industrialized desserts, industrialized sauces, boxed/canned juices, and powdered soft drinks, when compared to the North region. Data from the 2015 PeNSE study showed that adolescent students residing in the North and Midwest regions of the country had the highest prevalence of self-reported hunger [29]. Thus, the greater consumption in the North region may have economic motivations linked to the lower cost of UPFs when compared to food in general. 

Since 2016, there has been a weakening and dismantling of public policies that guarantee access to quality food. This scenario has contributed to an increase in poverty and worsening of the population’s living conditions. The 2017–2018 Brazilian Household Survey (Pesquisa de Orçamentos Familiares—POF) detected that 3.1 million families experienced hunger and by 2022, this number has increased alarmingly, with 33 million people living in this situation [30,31,32]. This scenario, mainly due to the COVID-19 pandemic, has exacerbated the world’s food crisis, mainly due to a reduction in family income resulting from social isolation that has led to reductions in salaries, workloads, temporary suspensions, and even layoffs [31] linked to food inflation. This crisis has increased the consumption of UPFs in this country, especially for people with less education [33], as observed in this study in terms of the consumption of the subgroups: sausages, industrialized desserts, industrialized sauces, chocolate drinks, boxed or canned juices, bread, soft drinks, and margarine. Therefore, investment in strategies to combat hunger and food insecurity is essential to ensure the consumption of food with nutritional quality. 

It is noted that the type of school, maternal education, race/skin color, and type of municipality can also be considered indirect indicators of the socioeconomic level of adolescents in this study. With an increase in income and the possibility of better food choices, these adolescents tend to have more balance in the principles that guide healthy eating: the supply of quality food, the availability of income in the hands of people, and the prices practiced in the market [34]. A higher chance of UPF consumption was found among adolescents who studied in private schools, such as crackers, cookies, soft drinks, margarine, boxed/canned juices, powdered refreshments, and flavored yogurt. A previous study carried out with PeNSE 2009, 2012, and 2015 [22,23,24] observed a decrease in the consumption of soft drinks and sweets over 6 years, for adolescents enrolled in both public and private schools [16]. The percentage observed in this study for soft drinks (40.53%) was higher than that reported in an article [16].

In our study, the students whose mothers had some level of access to education were less likely to consume ultra-processed foods than those whose mothers had no access to education. Regarding race/skin color, adolescents with a black skin color had a greater tendency to consume only industrialized desserts and chocolate drinks compared to white adolescents. From a cultural perspective, ethnicity has an important influence on individuals’ eating habits. In a society marked by racism, determining the life trajectory of individuals in relation to the environment in which they are inserted can be an indirect indicator of socioeconomic level, thus determining possible access to the principles that guide the balance for healthy eating [34,35]. 

Adolescence is marked by actions such as skipping breakfast [36,37], a preference for snacks over meals [38], the consumption of meals outside the home [39], the absence of parents/guardians at meals [40,41], few hours of sleep [42], a sedentary lifestyle [43], and an excessive exposure to screens [20,43]. These actions influence food consumption; although this was not evaluated in the present study, it is necessary to highlight these relationships. Living with parents may be related to the practice of having meals with parents. This practice is beneficial for adolescents, as a positive association was observed with a higher consumption of healthy eating markers and less unhealthy eating, contributing to an improvement in their health condition [44,45].

It should be recognized that this study has some limitations. This was a cross-sectional study, and for this reason, it did not infer causality. It relies on a self-administered structured questionnaire that was submitted to adolescents, and for this reason, it cannot provide a complex analysis of food consumption, but this approach has been previously validated and has often been used in epidemiological studies because of its low cost and breadth. The recrystallization of the samples was proposed for the analysis in this study, and they can be re-evaluated in future studies, depending on the nature of the investigation. The maternal educational level, which is considered a proxy for income, had a high percentage of non-knowledge among adolescents. Therefore, it is necessary to review the inclusion of this variable in future studies. 

We used data on the consumption of ultra-processed food from the previous day. Although it did not allow us to measure the quantity of food that had been consumed, the use of this method of assessing food intake may result in less memory bias when compared to food frequency questionnaires that assess the weekly or monthly consumption of foods. Moreover, there is no evidence of a greater potential bias in specific socioeconomic groups; therefore, the inequality values are not expected to be biased. 

## 5. Conclusions

Most adolescents reported the consumption of ultra-processed foods the day before the survey; this consumption demonstrates a great dependence on the UPF subgroups, especially crackers, cookies, bread, and soft drinks. Strategies to guarantee access to in natura and minimally processed foods must be part of the agenda of governments in various spheres to ensure the right to adequate and healthy food, added to food and nutrition education actions focusing on the adolescent public. 

## Figures and Tables

**Table 1:** Sociodemographic characteristics of Brazilian adolescents, according to the 2019 Brazilian School Health Survey, Brazil, 2019.

	%	IC 95%	
**Administrative dependence of the school**			
Public	85.14	84.50	85.76
Private	14.86	14.24	15.50
Total	100 (*n* = 159,245)		
**Gender**			
Male	49.17	48.48	49.86
Female	50.83	50.14	51.52
Total	100 (*n* = 158,799)		
**Race/skin color**			
White	35.72	34.94	36.51
Black	13.24	12.75	13.76
Asian	3.75	3.53	3.99
Mixed race	44.06	43.31	44.82
Indigenous	3.22	2.99	3.47
Total	100 (*n* = 155,806)		
**Region**			
North	10.62	9.87	11.42
Northeast	28.59	27.57	29.63
Southeast	39.09	37.74	40.45
South	13.58	12.84	14.36
Midwest	8.12	7.75	8.51
Total	100 (*n* = 159,245)		
**Type of municipality**			
Capital	22.84	22.09	23.62
Non Capital	77.16	76.38	77.91
Total	100 (*n* = 159,245)		
**Age**			
Less than 13	14.07	12.28	16.09
13 to 15 years old	51.26	49.17	53.34
16 or 17 years old	27.97	26.06	29.96
18 years old or more	6.70	6.04	7.43
Total	100 (*n* = 158,816)		
**Lives with mother**			
Yes	87.79	87.33	88.23
No	12.21	11.77	12.67
Total	100 (*n* = 159,155)		
**Lives with father**			
Yes	60.47	59.70	61.24
No	39.53	38.76	40.30
Total	100 (*n* = 159,107)		
**Maternal education level**			
No education	4.63	4.31	4.97
Incomplete elementary school	17.82	17.18	18.47
Complete elementary school	6.39	6.10	6.69
Incomplete high school	6.92	6.62	7.23
Complete high school	18.92	18.29	19.58
Incomplete higher education	5.73	5.44	6.04
Complete higher education	17.52	16.97	18.09
Do not know	22.06	21.34	22.79
Total	100 (*n* = 158,910)		

**Table 2:** Consumption of ultra-processed foods the day the before a survey of Brazilian adolescents, according to data from the 2019 Brazilian School Health Survey, Brazil, 2019.

	*n*	%	IC 95%	
Crackers	159,082	49.59	48.90	50.29
Cookies	159,075	46.66	46.04	47.28
Bread	159,073	41.75	41.12	42.38
Soft drinks	159,156	40.53	39.50	41.56
Margarine	159,074	40.05	39.19	40.92
Sausages	159,082	39.36	38.68	40.04
Industrialized desserts	159,067	33.27	32.36	34.20
Industrialized sauces	159,082	29.53	29.26	30.66
Chocolate drinks	159,114	25.76	25.19	26.35
Box/canned juices	159,112	24.89	24.35	25.43
Powdered refreshments	159,079	24.39	23.73	25.07
Ready-made meals	159,028	20.97	20.39	21.56
Flavored yogurts	159,100	16.89	16.38	17.41

**Table 3:** Results of Poisson regression models adjusted for the association between the consumption of ultra-processed foods on the previous day and the sociodemographic variables, according to the 2019 Brazilian School Health Survey, Brazil, 2019.

	Crackers		Cookies		Bread		Soft Drinks		Margarine	
	RP Adjusted **	IC 95% Min–Max	RP Adjusted **	IC 95% Min–Max	RP Adjusted **	IC 95% Min–Max	RP Adjusted **	IC 95% Min–Max	RP Adjusted **	IC 95% Min–Max
Type of school										
Public	1		1		1		1		1	
Private	1044 *	1034–1055	1026 *	1017–1035	0.981 *	0.973–0.989	1017 *	1004–1030	1047 *	1034–1060
Gender										
Male	1		1		1		1		1	
Female	0.992	0.984–1000	1008 *	1000–1016	1020 *	1011–1028	1019 *	1012–1027	0.986 *	0.979–0.993
Race/skin color										
White	1		1		1		1		1	
Black	0.967	0.953–0.981	0.971	0.956–0.985	1013	1000–1026	0.999	0.985–1014	0.968	0.957–0.979
Asian	0.968	0.944–0.922	0.990	0.969–1011	1007	0.987–1028	1008	0.988–1028	0.972	0.954–0.991
Mixed race	0.976	0.966–0.985	0.989	0.980–0.998	1012	1004–1021	1011	1001–1020	0.978	0.970–0.985
Indigenous	0.981	0.959–1003	0.992	0.971–1013	1001	0.982–1021	0.999	0.979–1018	0.995	0.975–1016
Region										
North	1		1		1		1		1	
Northeast	0.980 *	0.965–0.955	0.949 *	0.937–0.960	1018 *	1004–1033	0.999	0.980–1020	1006	0.991–1020
Southeast	1039 *	1022–1056	0.989	0.977–1002	1029 *	1013–1045	0.945 *	0.924–0.967	1084 *	1067–1101
South	1073 *	1054–1091	1027 *	1013–1042	0.918 *	0.902–0.935	0.961 *	0.940–0.983	1103 *	1084–1122
Midwest	1037 *	1021–1054	1015 *	1003–1028	1031 *	1015–1047	0.945 *	0.924–0.966	1098 *	1082–1115
Type of municipality										
Capital	1		1		1		1		1	
Non-capital	0.991 *	0.982–0.999	0.998	0.989–1007	1013 *	1006–1020	1019 *	1008–1031	1042 *	1033–1051
Age (years)										
Less than 13	1		1		1		1		1	
13 to 15	1016 *	1001–1030	0.994	0.980–1009	0.990	0.978–1003	0.996	0.978–1015	0.980 *	0.967–0.992
16 or 17	1055 *	1039–1071	1020 *	1005–1036	0.994	0.980–1008	1000	0.980–1021	0.988	0.972–1003
18 or more	1050 *	1029–1072	1025 *	1005–1045	1000	0.983–1018	1009	0.986–1032	0.984	0.964–1003
Lives with mother										
Yes	1		1		1		1		1	
No	1001	0.990–1013	1006	0.994–1019	1009	0.997–1021	0.986 *	0.973–0.998	1001	0.989–1013
Lives with father										
Yes	1		1		1		1		1	
No	0.992	0.984–1000	1003	0.995–1011	0.999	0.991–1007	1003	0.996–1011	0.991 *	0.983–0.998
Maternal education level										
No education	1		1		1		1		1	
IES ***	0.997	0.977–1016	0.982	0.963–1002	0.969 *	0.956–0.983	0.981 *	0.964–0.997	0.970 *	0.953–0.987
CES ***	0.977	0.955–1000	0.970 *	0.948–0.993	0.954 *	0.936–0.972	0.974 *	0.954–0.994	0.960 *	0.940–0.980
IHS ***	1000	0.977–1024	0.983	0.963–1004	0.940 *	0.923–0.957	0.963 *	0.944–0.982	0.962 *	0.942–0.983
CHS ***	1010	0.990–1031	0.996	0.977–1016	0.941 *	0.927–0.955	0.950 *	0.933–0.967	0.965 *	0.949–0.981
IHE ***	1000	0.977–1024	0.982	0.959–1005	0.938 *	0.918–0.959	0.947 *	0.926–0.969	0.983	0.962–1004
CHE ***	1013	0.992–1034	1006	0.986–1026	0.936 *	0.921–0.952	0.948 *	0.930–0.966	0.985	0.968–1003

* *p* < 0.05, Poisson regression model. ** Ultra-processed food adjusted for sociodemographic variables (school type, gender, race/skin color, region, type of municipality, age, lives with mother, lives with father, maternal education level). *** IES: incomplete elementary school; CES: complete elementary school; IHS: incomplete high school; CHS: complete high school; IHE: incomplete higher education; CHE: complete higher education.

**Table 4:** Results of Poisson regression models adjusted for the association between the consumption of ultra-processed foods on the previous day and the sociodemographic variables, according to the 2019 Brazilian School Health Survey, Brazil, 2019.

	Sausages		Industrialized Desserts		Industrialized Sauces		Chocolate Drinks		Boxed/Canned Juices	
	RP Adjusted **	IC 95% Min–Max	RP Adjusted **	IC 95% Min–Max	RP Adjusted **	IC 95% Min–Max	RP Adjusted **	IC 95% Min–Max	RP Adjusted **	IC 95% Min–Max
Type of school										
Public	1		1		1		1		1	
Private	1001	0.992–1011	0.958 *	0.947–0.970	0.997	0.988–1005	0.983 *	0.976–0.990	1064 *	1056–1072
Gender										
Male	1		1		1		1		1	
Female	1010 *	1002–1018	0.956 *	0.950–0.962	1002	0.996–1009	1010 *	1004–1017	1003	0.996–1010
Race/skin color										
White	1		1		1		1		1	
Black	0.995	0.983–1008	1033 *	1021–1045	1009	0.996–1023	1012 *	1002–1023	0.975 *	0.964–0.987
Asian	0.993	0.973–1014	1024 *	1007–1041	1011	0.992–1,029	1012	0.994–1030	0.981 *	0.964–0.999
Mixed race	1000	0.991–1009	1022 *	1013–1031	1013 *	1006–1021	1008 *	1001–1016	0.985 *	0.978–0.992
Indigenous	1011	0.991–1031	1044 *	1025–1065	1011	0.990–1031	0.990	0.972–1008	0.981 *	0.963–0.999
Region										
North	1		1		1		1		1	
Northeast	0.981 *	0.971–0.991	0.991	0.979–1004	0.990	0.979–1000	1004	0.995–1014	0.996	0.988–1005
Southeast	0.958 *	0.946–0.970	0.937 *	0.921–0.954	0.952 *	0.939–0.964	0.938 *	0.928–0.948	0.941 *	0.931–0.952
South	0.933 *	0.922–0.944	0.944 *	0.927–0.961	0.922 *	0.910–0.935	0.950 *	0.937–0.963	0.927 *	0.915–0.938
Midwest	0.979 *	0.968–0.989	0.947 *	0.933–0.960	0.964 *	0.954–0.975	0.960 *	0.950–0.969	0.966 *	0.957–0.975
Type of municipality										
Capital	1		1		1		1		1	
Non-capital	1008 *	1000–1016	1013 *	1004–1023	1006	0.999–1013	1006 *	1000–1011	1005	0.998–1012
Age (years)										
Less than 13	1		1		1		1		1	
13 to 15	0.987 *	0.976–0.998	1003	0.990–1016	0.987 *	0.976–0.998	1018 *	1006–1030	0.986 *	0.977–0.995
16 or 17	0.993	0.982–1004	1018 *	1003–1033	0.974 *	0.963–0.986	1042 *	1031–1054	1003	0.993–1013
18 or more	1008	0.993–1022	1025 *	1007–1043	0.982 *	0.966–0.997	1051 *	1037–1067	0.998	0.985–1012
Lives with mother										
Yes	1		1		1		1		1	
No	0.997	0.983–1010	1005	0.995–1015	1000	0.989–1011	1004	0.994–1014	1005	0.995–1015
Lives with father										
Yes	1		1		1		1		1	
No	0.996	0.989–1003	1009 *	1002–1016	0.998	0.991–1005	1008 *	1002–1014	0.991 *	0.985–0.998
Maternal education level										
No education	1		1		1		1		1	
IES ***	0.972 *	0.955–0.989	0.974 *	0.961–0.987	0.961 *	0.947–0.974	0.973 *	0.963–0.984	0.987	0.975–1000
CES ***	0.960 *	0.941–0.980	0.956 *	0.941–0.972	0.943 *	0.928–0.958	0.953 *	0.940–0.967	0.982 *	0.966–0.998
IHS ***	0.963 *	0.944–0.982	0.962 *	0.946–0.978	0.939 *	0.925–0.953	0.956 *	0.942–0.970	0.980 *	0.965–0.995
CHS ***	0.962 *	0.944–0.980	0.944 *	0.930–0.959	0.930 *	0.915–0.944	0.954 *	0.944–0.964	0.989	0.976–1003
IHE ***	0.979 *	0.960–0.999	0.941 *	0.924–0.958	0.927 *	0.909–0.944	0.932 *	0.919–0.945	0.993	0.976–1010
CHE ***	0.975 *	0.958–0.992	0.927 *	0.913–0.941	0.926 *	0.911–0.941	0.932 *	0.924–0.948	1012	0.998–1023

* *p* < 0.05, Poisson regression model. ** Ultra-processed food adjusted for sociodemographic variables (school type, gender, race/skin color, region, type of municipality, age, lives with mother, lives with father, maternal education level). *** IES: incomplete elementary school; CES: complete elementary school; IHS: incomplete high school; CHS: complete high school; IHE: incomplete higher education; CHE: complete higher education.

**Table 5:** Results of Poisson regression models adjusted for the association between the consumption of ultra-processed foods on the previous day and the sociodemographic variables, according to the 2019 Brazilian School Health Survey, Brazil, 2019.

	Powdered Refreshments		Ready-Made Meals		Flavored Yogurts	
	RP Adjusted **	IC 95% Min–Max	RP Adjusted **	IC 95% Min–Max	RP Adjusted **	IC 95% Min–Max
**Type of school**						
Public	1		1		1	
Private	1064 *	1056–1072	1032	1026–1039	1012 *	1006–1018
**Gender**						
Male	1		1		1	
Female	1003	0.996–1010	0.995	0.989–1000	1008 *	1003–1013
**Race/skin color**						
White	1		1		1	
Black	0.975 *	0.964–0.987	0.982	0.973–0.991	1002	0.993–1010
Asian	0.981 *	0.964–0.999	0.986	0.972–1001	0.996	0.983–1008
Mixed race	0.985 *	0.978–0.992	0.991	0.985–0.998	1001	0.995–1007
Indigenous	0.981 *	0.963–0.999	0.981	0.965–0.996	0.993	0.978–1009
**Region**						
North	1		1		1	
Northeast	0.996 *	0.988–1005	0.999	0.989–1008	1009 *	1002–1016
Southeast	0.941 *	0.931–0.952	1008	0.997–1018	0.999	0.991–1007
South	0.927 *	0.915–0.938	0.996	0.984–1009	0.997	0.988–1007
Midwest	0.966 *	0.957–0.975	1016	1007–1026	1004	0.997–1013
**Type of municipality**						
Capital	1		1		1	
Non Capital	1005	0.998–1012	0.997	0.992–1003	1005 *	1000–1010
**Age**						
Less than 13	1		1		1	
13 to 15	0.986 *	0.977–0.995	1007	0.998–1016	1024 *	1013–1036
16 or 17	1003	0.993–1013	1027	1017–1037	1040 *	1028–1052
18 or more	0.998	0.985–1012	1020	1007–1034	1040 *	1026–1055
**Lives with mother**						
Yes	1		1		1	
No	1005	0.995–1015	0.993	0.984–1003	1002	0.994–1010
**Lives with father**						
Yes	1		1		1	
No	0.991 *	0.985–0.998	0.993	0.987–0.999	1005	0.999–1010
**Maternal education level**						
No education	1		1		1	
IES ***	0.987	0.975–1000	1009	0.995–1023	1007	0.997–1017
CES ***	0.982 *	0.966–0.998	1013	0.994–1031	0.996	0.983–1009
IHS ***	0.980 *	0.965–0.995	1009	0.992–1027	1008	0.995–1020
CHS ***	0.989	0.976–1003	1027	1013–1042	0.995	0.985–1005
IHE ***	0.993	0.976–1010	1026	1009–1043	0.996	0.981–1011
CHE ***	1012	0.998–1027	1019	1005–1003	0.989	0.978–1001

* *p* < 0.05, Poisson regression model. ** Ultra-processed food adjusted for sociodemographic variables (school type, gender, race/skin color, region, type of municipality, age, lives with mother, lives with father, maternal education level). *** IES: incomplete elementary school; CES: complete elementary school; IHS: incomplete high school; CHS: complete high school; IHE: incomplete higher education; CHE: complete higher education.

## Data Availability

Publicly available datasets were analyzed in this study. This data can be found here: https://www.ibge.gov.br/estatisticas/sociais/populacao/9134-pesquisa-nacional-de-saude-do-escolar.html?edicao=31442&t=downloads.
